# Supplementary figures and images for: Mining folded proteomes in the era of accurate structure prediction
Source: PLoS Comput Biol. 2022 Mar 25;18(3):e1009930. doi: 10.1371/journal.pcbi.1009930 (PMC8986115; doi:10.1371/journal.pcbi.1009930)

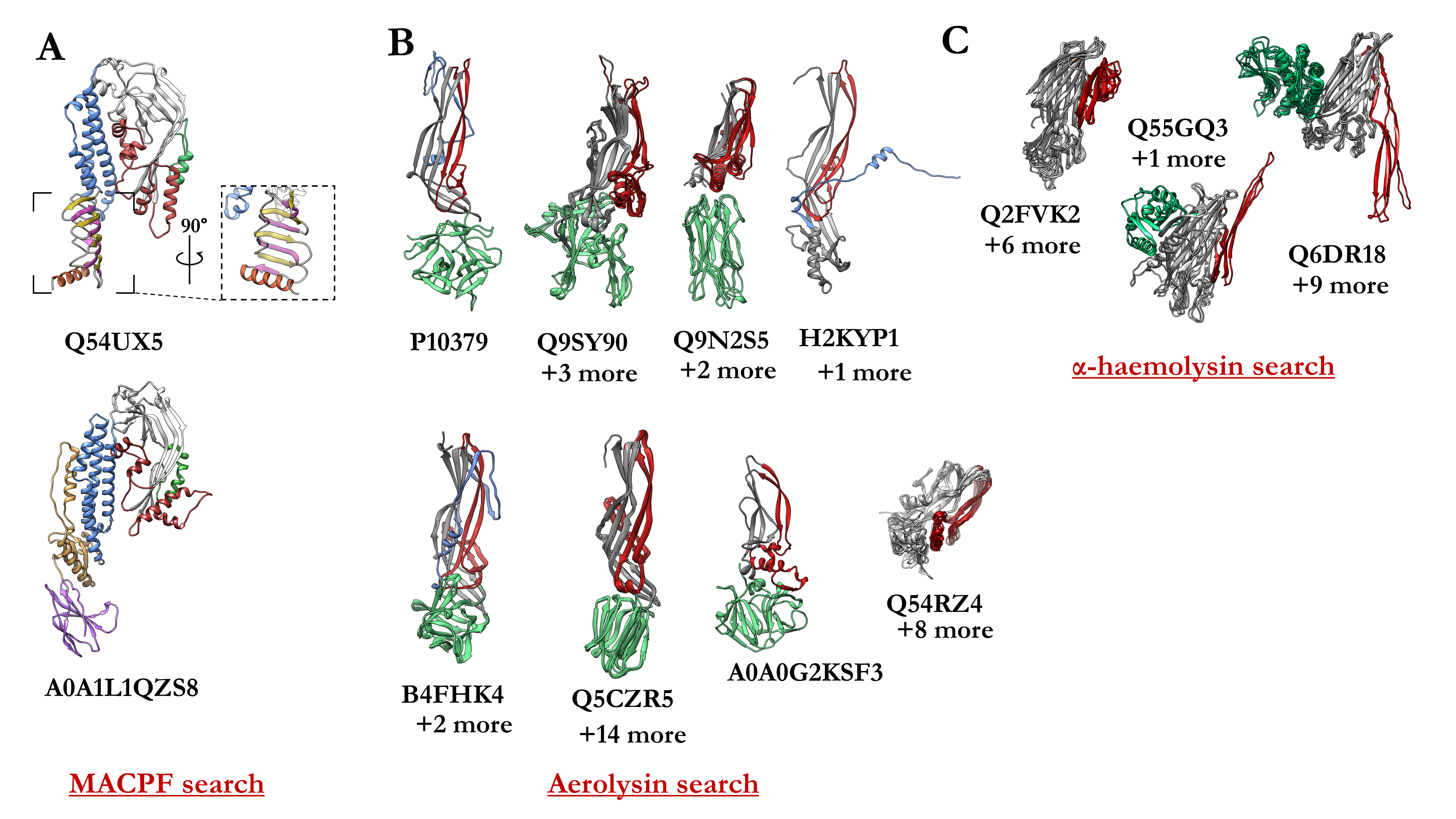

Supplement: S1 Fig — a. Identified MACPFs from slime mold and zebrafish. b. Various β-PFPs with aerolysin-like pore-forming domains which resemble aerolysin, lysenin, epsilon toxin, monalysin and LSL. Observed in numerous organisms including drosophila, C. elegans, zebrafish, yeast, among others. c. Several novel α-haemolysin-like proteins identified in S. aureus and plants. The putative receptor binding domain is coloured green and the pore-forming domain is coloured grey with the expected transmembrane region in red. (TIF) [file pcbi.1009930.s001.TIF]
