# Supplementary material for: Mining folded proteomes in the era of accurate structure prediction
Source: PLoS Comput Biol. 2022 Mar 25;18(3):e1009930. doi: 10.1371/journal.pcbi.1009930 (PMC8986115; doi:10.1371/journal.pcbi.1009930)
Supplement: S1 Text — (DOCX) [file pcbi.1009930.s004.docx]

**Supplementary Note 1: Definition of a “foldome”**

A single structural state of a molecule represents one configuration from the abstract conformational space in which all possible atomic arrangements exist. Similarly, a structural ensemble therefore corresponds to a set that samples from the conformational space. We refer the reader to the elegant discussion of “the structure” by Abbas Ourmazd [1].

Likewise, we consider the foldome (an “entire set of folds”) to be a complete set of all protein structures and conformations for a given proteome. This is akin to an abstract space that consists of many conformational/configurational subspaces (one for each protein member).

Since the AlphaFold predictions provide only a single conformational prediction per protein, these sets can be thought of as sampling from the foldome – one state for each molecule. Therefore, these are not complete sets and only coarsely approximate the true foldome of a given organism.

Therefore, we caveat our use of the term “foldome” by acknowledging these sets are not true, complete foldomes. But rather, a first pass approximation of the foldome – similar to early attempts to sequence and quantify the human genome.

Future releases of AlphaFold, as well as additional experimentally determined structures, will continue to populate our picture of the foldome.

1. Ourmazd A. Cryo-EM, XFELs and the structure conundrum in structural biology. Vol. 16, Nature Methods. 2019. p. 941–4.
