# Supplementary material for: Mining folded proteomes in the era of accurate structure prediction
Source: PLoS Comput Biol. 2022 Mar 25;18(3):e1009930. doi: 10.1371/journal.pcbi.1009930 (PMC8986115; doi:10.1371/journal.pcbi.1009930)
Supplement: Attachment — Submitted filename: response_to_reviewers.docx [file pcbi.1009930.s005.docx]

Specifically, please provide a command line tool or ideally a web server to perform the structural similarity search and analysis from the paper.

We were unable to secure financial support to build and host a web server for the full AlphaFold search described in our manuscript. This is largely due to the high computational requirements of several hundred CPU hours per search, which made hosting a service for 100-1000 users per month unfeasible (we note the DALI server cluster typically conducts over 100 searches per day). Fortunately, during review, the DALI server (hosted by Helsinki University, OLM group, Biocenter Finland) was modified to enable single-proteome searches against the AlphaFold database.

This meets the requirements of most new users, however a full, extensive search of the entire AlphaFold database is not yet supported (likely also due to the high computational cost). For these purposes, a pre-processed AlphaFold database has also been provided that is compatible with DALI, this greatly facilitates DALI based searches. Information and command line tools are provided alongside the DALI code: <http://ekhidna2.biocenter.helsinki.fi/dali/digest.html>. We did not seek to duplicate these efforts.

**Reviewer's Responses to Questions**

**Comments to the Authors:**

Please note here if the review is uploaded as an attachment.

**Reviewer #1:**

The manuscript presents a case study on mining the computationally predicted structures in the recently released AlphaFold Protein Structure Database for structural similarities. The authors use established fold recognition algorithms to compare proteins from the entire database to known members of several pore-forming protein families and succeed in identifying previously unknown members of such families.

The authors' methods represent a clever and straightforward-to-apply workflow to explore computationally predicted structures. The paper explains well why the particular results from their examples are of biological interest. Beyond corroborating the approach itself, these results demonstrate that computationally predicted protein structures can be used to discover relevant connections and relations between different proteins that cannot be obtained from sequence databases.

This study is the first of its kind (as far as I am aware) which comes with a lot of merit but also necessitates some additional tests and discussion to form a solid basis for future similar ventures. In particular, the following points should be addressed:

- It is not clear to me whether using a different reference protein from one family would lead to the same result. For example, if instead of GSDM-D one had searched for similarities to a MACPF, would the results have included C11orf42, too? Similarly, could one identify all MACPF/GSDM members by using C11orf42 as a reference?

Generally speaking, DALI is robust to the chosen search probe and it does not typically affect the results.

While we only performed one GSDM search, we conducted several searches using monomers of different MACPF family members (MPEG1, perforin, C9). In these analyses, we frequently saw the same members being identified from the AlphaFold database, irrespective of the probe. Notably, C9 is in the extended conformation, unlike PRF and MPEG1, but was still sufficient to identify other MACPF members. These experiments also frequently identified GSDMs.

We have added a clarification in the manuscript.

“Repeating this search with different MACPF probes (e.g., MPEG1, perforin, C9) yields very similar results, indicating DALI is robust to the chosen search template.”

- When expanding the analysis to all proteomes (line 52), the computational cost increases strongly. Could one in principle alleviate this problem with a less expensive pre-screen that excludes proteins that, by some simpler criteria, clearly cannot have a similar fold?

Yes, in principle. We have added some more discussion of future work that may be beneficial in this regard. We are also exploring different ways to improve scalability of the analysis. Namely, porting DALI code to modern python, re-visiting GPU acceleration and exploring new parallelisation schemes to take advantage of distributed computing and HPC infrastructure.

“The need for modern and sensitive implementations of fold-matching algorithms has once again become relevant. The current release of AlphaFold predictions has expanded the available structural database by more than double, with additional contributions expected to reach nearly a million entries in the near future. Exhaustive search algorithms, such as DALI, are slow and scale poorly, meaning searching structural databases of these sizes is not tractable. As such improvements and further work on fold matching algorithms is paramount to enable rapid searching and exploration of these new resources. Other algorithms for fold matching are notably much faster than DALI, such as 3D Zernike moment decomposition of protein structures and subsequent k-means nearest neighbour29. However, these come with sensitivity trade-offs. A mixed approach may be beneficial, where 3D Zernike descriptors could be initially used to filter the database, followed by an exhaustive DALI search. This would be akin to a coarse initial pass to define a smaller subset of the search space to allow a computationally tractable exhaustive local search. Alternatively, sequences could be first filtered based on exclusion criteria, such as length.”

The authors introduce the term "foldome" for a set of structures corresponding to a proteome. According to their definition (one of many potential folds per sequence), a proteome has multiple potential foldomes. This definition leaves no room to describe a set of structural ensembles, which would intuitively be associated with the term foldome meaning "entire set of folds."

We have added a supplementary note in the manuscript to clarify our use of the term. We define “foldome” and acknowledge a caveat in its use.

“A single structural state of a molecule represents one configuration from the abstract conformational space in which all possible atomic arrangements exist. Similarly, a structural ensemble therefore corresponds to a set that samples from the conformational space. We refer the reader to the elegant discussion of “the structure” by Abbas Ourmazd^32^.

Likewise, we consider the foldome (an “entire set of folds”) to be a complete set of all protein structures and conformations for a given proteome. This is akin to an abstract space that consists of many conformational/configurational subspaces (one for each protein member).

Since the AlphaFold predictions provide only a single conformational prediction per protein, these sets can be thought of as sampling from the foldome – one state for each molecule. Therefore, these are not complete sets and only coarsely approximate the true foldome of a given organism.

Therefore, we caveat our use of the term “foldome” by acknowledging these sets are not true, complete foldomes. But rather, a first pass approximation of the foldome – similar to early attempts to sequence and quantify the human genome.

Future releases of AlphaFold, as well as additional experimentally determined structures, will continue to populate our picture of the foldome.”

- While the "era of accurate structure prediction" is indubitably owed to the machine learning techniques, as much credit should be attributed to advances in the experimental techniques that determine the structures from which these models learn.

We strongly agree, we have modified the text to emphasis this point.

“By virtue of advances in experimental techniques and judicious deposition of results over several decades, this limitation is now being addressed by machine learning approaches.”

- The paper alludes to "more comprehensive evolutionary analyses" in line 59. It would help to elaborate on that or provide a few examples.

We had added an example in the text.

“For example, by using newly identified structural homologs as seed sequences for iterative PSI-BLAST searches of sequence databases or by studying the gene loci of newly discovered family members.”

**Reviewer #2:**

**Summary of the paper**

The authors point out a fairly obvious use-case for ML predictions of protein structure and then describe some interesting applications. Structures predicted by AlphaFold2 or similar neural networks are queried using fold recognition tools such as DALI in order to find structural homologs with very low sequence identity and impute function to uncharacterized proteins. The primary contributions are:

- The authors identify new pore-forming proteins, including a new human perforin / GSDM with only 1% sequence homology to known examples

- The authors query human proteins with confident structural predictions against the remainder of the AF2 human foldome, identifying possible functions for several uncharacterized proteins

- The authors query predicted structures for Pfam domains of unknown function against the S. aureus AF2 foldome

**Main Review**

**Strengths**

In general, the paper is concise and clear.

I appreciated the discussion of limitations and possible improvements

The example of structural homology with only 1% sequence homology is impressive!

**Major Weaknesses**

I think the contribution would be stronger if the authors also made it easier (with a command-line tool or web server) for scientists with less-specialized knowledge to do structural homology search against AF2 predictions.

Since submission the DALI team have updated their server to facilitate much of the workflow and analysis we have performed. There remain limitations, however scientists with less-specialised knowledge should have sufficient access to these methods.

**Minor weaknesses**

The paper could be stronger with justifications or reasoning for tool choices. Why use DALI vs SA Tableau for different tasks?

DALI is a notable choice as it is widely accepted to be the most robust and sensitive method – however as we discuss in the manuscript it is computationally expensive. SA Tableau is GPU accelerated, which makes performing exhaustive all-vs-all comparisons of proteomes to reference domain libraries computationally tractable. We have now added some discussion about alternatives.

“We employ the popular DALI algorithm due to its sensitivity and robustness.”

“Here, we employed a GPU-accelerated fold recognition software, SA Tableau search^26^, to expedite the large comparison which was not computationally tractable with DALI.”

Or why use trRosetta predictions instead of AF2 or RosettaFold predictions for some tasks?

The use of trRosetta predictions was purely a choice of convenience. As these structures represent all the known protein families already grouped into a single library. For all other purposes the use of AlphaFold2 predictions were used (once again due to convenience as the database is publicly available and pre-computed).

We have modified the manuscript as follows:

“The public nature of these representative structures makes the trRosetta models a convenient choice, however, computing AlphaFold predictions for each Pfam entry would likely give an improved representative library.”

Likewise, how were the foldomes chosen?

Where possible, all available foldomes were used. These foldomes correspond to particular organisms for which the proteomes are well defined and verified.

In the case of specific analyses on the human foldome, this simply reflects the relevance to human disease. As uncharacterised proteins within the human proteome represent yet to be studied entities that may have implications for human biology.

Why limit searches to the human foldome or the S. aureaus foldome?

Performing an all-vs-all comparison of the trRosetta library against a whole foldome corresponds to over 20 million comparisons. This was the smallest and therefore most computationally tractable foldome to choose for the purposes of an example.

“for simplicity we chose the smallest available foldome”

Would it be better to search against a deduplicated, "representative" predicted structure library? If not, why not?

The use of trRosetta is already a “representative” and deduplicated set of protein families, similarly each proteome (as of current) has only a single predicted structure per protein member. As such these databases are currently non-redundant.

However, in the future, additional predictions in distinct conformational states may create duplicate entries. These would not be easily deduplicated, as the search-and-match algorithm is sensitive to tertiary protein fold and it is not clear *a priori* how to discard one of two distinct and equally valid conformations.

We have added a new discussion paragraph to suggest alternative computational strategies for searching large databases.

“The need for modern and sensitive implementations of fold-matching algorithms has once again become relevant. The current release of AlphaFold predictions has expanded the available structural database by more than double, with additional contributions expected to reach nearly a million entries in the near future. Exhaustive search algorithms, such as DALI, are slow and scale poorly, meaning searching structural databases of these sizes is not tractable. As such improvements and further work on fold matching algorithms is paramount to enable rapid searching and exploration of these new resources. Other algorithms for fold matching are notably much faster than DALI, such as 3D Zernike moment decomposition of protein structures and subsequent k-means nearest neighbour29. However, these come with sensitivity trade-offs. A mixed approach may be beneficial, where 3D Zernike descriptors could be initially used to filter the database, followed by an exhaustive DALI search. This would be akin to a coarse initial pass to define a smaller subset of the search space to allow a computationally tractable exhaustive local search. Alternatively, sequences could be first filtered based on exclusion criteria, such as length.”

While acknowledging that bench verification of imputed functions is out of scope for this paper, I would like some discussion of the trustworthiness of functions imputed via structural homology, even under the assumption that the structure predictions are accurate.

We have added some additional discussion on this matter. We believe that both fold-matching and primary sequence analysis techniques will be highly complementary.

“Unlike domain assignment by primary sequence analysis, fold-matching algorithms are sensitive and robust. We anticipate that domain assignment by fold-matching will likely provide more accurate and informative predictions over existing sequence analysis methods, especially in contexts where sequences have poor overall homology or possess discontinuous breaks and insertions. Of course, imputed function remains to be experimentally validated.”

Given that AF2 predicts accurate protein structures given MSAs, could we do homology search by just comparing MSAs?

In general, this is the case and has been performed for many years. However, sequences with weak homology often do not have sufficient signal in multiple sequence alignments to confidently assign domains. This is notable in the example of C11orf42, for which a clear GSDM-like domain is present but for which only 1% sequence homology is present. This has not been detected previously by sequence searches alone.

Often discontinuous breaks or insertions into domains (such as the loop insertion in C12orf56, figure 2D) will obfuscate multiple sequence alignments and prevent correct assignment of domains. In this contexts, structure based identification is superior.

**Summary of the review**

While the idea of applying structural homology search to ML structure predictions is fairly obvious, the authors do it carefully and find convincing, biologically-interesting results. Together, this makes a strong case for making this sort of search a part of the standard toolbox when working with proteins of unknown function, and the work is of broad interest to protein biologists and engineers. Overall, this paper is suitable publication in PLOS Comp Bio with minor revisions.
